# Enhancement of First Hydrogenation of Ti_1_V_0.9_Cr_1.1_ BCC Alloy by Cold Rolling and Ball Milling

**DOI:** 10.3390/ma13143106

**Published:** 2020-07-12

**Authors:** Salma Sleiman, Anis Aliouat, Jacques Huot

**Affiliations:** 1 Hydrogen Research Institute, Université du Québec à Trois-Rivières, 3351 des Forges, Trois-Rivières, QC G9A 5H7, Canada; salma.sleiman@uqtr.ca; 2 Faculty of Sciences, Université de Poitiers, 15, rue de l’Hôtel Dieu—TSA 71117, 86073 Poitiers CEDEX 9, France; aliouat.anis78@gmail.com

**Keywords:** BCC alloys, kinetics, cold rolling, ball milling, hydrogen storage

## Abstract

In this study, we evaluated the effects of a mechanical treatment by cold rolling (CR) and ball milling (BM) on the first hydrogenation of Ti_1_V_0.9_Cr_1.1_ alloy. The as-cast alloy has a body-centered cubic (BCC) crystal structure, and the first hydrogenation at room temperature under 20 bars of hydrogen is practically impossible. However, the samples mechanically activated by CR or BM readily absorbed hydrogen. The sample cold-rolled for one pass exhibited faster kinetics than the sample ball-milled for five minutes, but both samples reached the same storage capacity of 3.6 wt % hydrogen. Increasing the amount of rolling or the milling time decreased the hydrogen capacity. CR is considered the best and most efficient method for the activation of Ti_1_V_0.9_Cr_1.1_ alloy.

## 1. Introduction

Metal hydrides are considered reliable and safe materials for storing hydrogen at a reasonable temperature and pressure, with relatively low cost and high hydrogen storage volumetric density [1,2,3]. Among them, the Ti–V–Cr system has been intensively studied as an attractive material for hydrogen storage due to its relatively high absorption capacity of up to 3.7 wt % in mild conditions [4,5,6]. The Ti–V–Cr system has tunable hydrogen sorption properties due to the wide range of possible chemical compositions [7,8,9]. For instance, the cyclic performance of Ti_21.5_V_40_Cr_38.5_ alloy was found to be more durable than that of Ti_32_V_20_Cr_48_ [8]. Lin et al. found that the Ti_0.8_Cr_1.2_V alloy have better cyclic hydrogen properties than TiCrV alloy [10]. Another example is (TiCr_1.8_)_0.6_V_0.4_, which has the highest reversible hydrogen capacity among the series of (TiCr_1.8_)_1−*x*_V*_x_* alloys with *x* = 0.2, 0.6, and 0.8 [9]. However, the Ti–V–Cr system shows slow hydrogen sorption kinetics, and the first hydrogenation is usually difficult [11]. From a practical point of view, the first hydrogenation (also called activation) is an important aspect. It may require high pressure and temperature and thus increases the cost of the hydride. The sluggish first hydrogenation is usually explained by the presence of a surface oxide that blocks hydrogen diffusion. Generally, to force hydrogen through the oxide layers, activation at high temperature and high hydrogen pressure is needed [12,13]. There is no formal method to determine the temperature and pressure of activation. For this reason, we decided to conduct the first hydrogenation at room temperature and 20 bars of hydrogen. These are the usual working conditions of the alloys.

Alloying Ti–V–Cr alloys with Zr [14,15,16] or Zr_7_Ni_10_ [17,18] results in improvement of the activation and kinetics of absorption. This improvement has been explained by the appearance of a secondary phase, which acts as a gateway for hydrogen to reach the main phase. Heat treatment could also enhance the first hydrogenation by changing the alloy’s microstructure [19,20,21]. Nanocrystallinity, achieved by ball milling (BM), improves the activation kinetics [22,23,24], potentially related to the increase in interfaces and the specific surface area [25]. Small particle size also beneficial affects activation [26]. In a theoretical investigation, Shelyapina et al. showed that the disordered BCC solid solution is more stable than the ordered AB_2_ compound [27]. Thus, we expected that our Ti–V–Cr alloy would have the BCC structure, and the full hydride phase would be the face-centered cubic (FCC) structure.

Mechanical deformation techniques including BM, cold rolling (CR), high-pressure torsion (HPT), and equal channel angular pressure (ECAP) are efficient in reducing crystallite size and introducing defects in materials, which improve the hydrogenation kinetics [28]. Among these deformation techniques, CR can be considered more promising from an industrial point of view due to its simplicity and easy scalability [29,30,31,32]. In addition, CR is an efficient method for regenerating air-exposed samples [33,34].

In a previous investigation, we showed that Ti_1_V_0.9_Cr_1.1_ alloy can be easily activated when a small amount of Zr (4 wt %) is added to the melt. This easy activation was attributed to the presence of a Zr-rich secondary phase [14]. Here, we aimed to see if mechanical treatment could be as effective as the addition of Zr for the enhancement of first hydrogenation. Thus, the Ti_1_V_0.9_Cr_1.1_ alloy was processed using CR and BM, and the effects of mechanical deformation on the first hydrogenation and microstructure investigated.

## 2. Materials and Methods

The raw materials Ti sponge (99.95%), V pieces (99%), and Cr pieces (99%) were purchased from Alfa-Aesar and used without further purification. The alloy was prepared by arc melting after mixing all the raw elements in the desired proportion. The melting was performed under 0.7 bars of argon. Each pellet was melted, turned over, and melted three times again to ensure homogeneity. The pellet was then hand crushed using a hardened steel mortar and pestle in an argon-filled glovebox. The CR apparatus used was a Durston DRM 130 (High Wycombe, UK) that was modified so the powder samples could be rolled vertically. Rolling experiments were performed in air by inserting the powder obtained from hand crushing between two 316 stainless steel plates to prevent contamination from the rolls. Rolling was performed once, three times, and six times. After the first roll, the powder consolidated on a plate. The thickness of the plate was about 0.5 mm. This plate was then folded in two and rolled again to the final number of rollings. Thus, a thickness reduction of around 50% was obtained after each pass.

BM was carried out on a Spex 8000 high energy ball mill (SPEX SamplePrep, Metuchen, NJ, USA) in a hardened 55 cc steel crucible and balls with a powder-to-ball mass ratio of 1/10. All loadings and unloading of powder in the crucible were performed in an argon-filled glove box. BM was performed for 5, 15, 30, and 60 min (BM 5min, BM 15min, BM 30min, and BM 60 min, respectively) with a vibration frequency of 1060 cycles per minute. After the milling process, the powders were removed from the crucible in the glove box. The crystal structure was determined by X-ray diffraction (XRD) using a Bruker D8 Focus (Bruker AXS LLC, Madison, WI, USA) with Cu-Kα radiation. Lattice parameters were evaluated from Rietveld refinement using Topas software (V6.0, Bruker AXS LLC, Madison, WI, USA) [35]. Microstructure was investigated using a Hitachi Su1510 scanning electron microscopy (Hitachi High-Tech America, Dallas, TX, USA). First, hydrogenation was performed at room temperature (25 °C) under 20 bars of hydrogen pressure using a homemade Sievert’s apparatus. The samples were filled in the reactor and kept under a dynamic vacuum at room temperature for 1 h before the measurement. After reaching full hydrogenation, the absorption experiment was stopped, keeping the sample under hydrogen pressure. After that, the sample was taken for XRD measurement in the air at room temperature and atmospheric pressure.

## 3. Results and Discussion

### 3.1. Morphology

**Figure 1:**
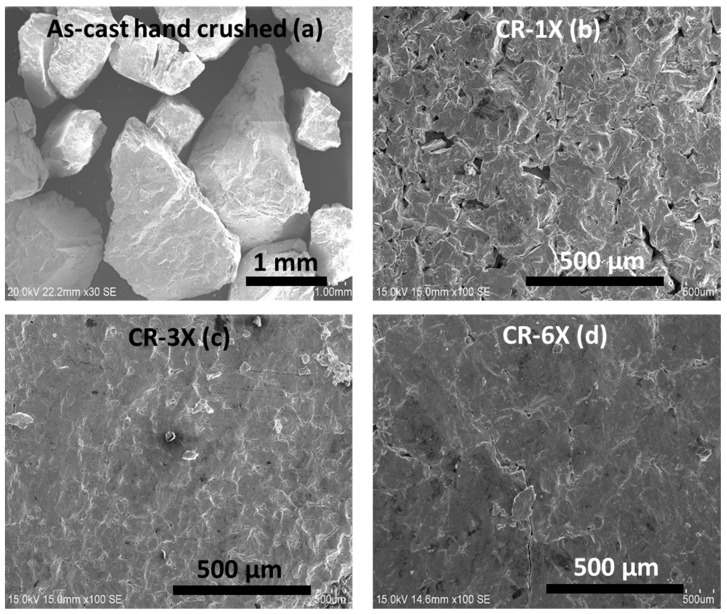
Scanning electron microscopy (SEM) micrographs of hand crushed (**a**), CR-1X (**b**), CR-3X (**c**), and CR-6X (**d**) of Ti_1_V_0.9_Cr_1.1_ alloy.

Figure 1 shows the morphologies of Ti_1_V_0.9_Cr_1.1_ alloy in the as-cast state after hand crushing and CR.

The as-cast sample, after hand crushing, consisted of particles ranging from 0.65 to 2.2 mm. After CR, the sample showed consolidation of the powder. CR the sample one time agglomerated the powder into plates but with some voids, as presented in Figure 1b. The plates seemed to be porous. After three times of CR, the voids within the plates disappeared, but some cracks remained. With six rolling passes, the plate seemed to be more consolidated.

### 3.2. Crystal Structure

**Figure 2:**
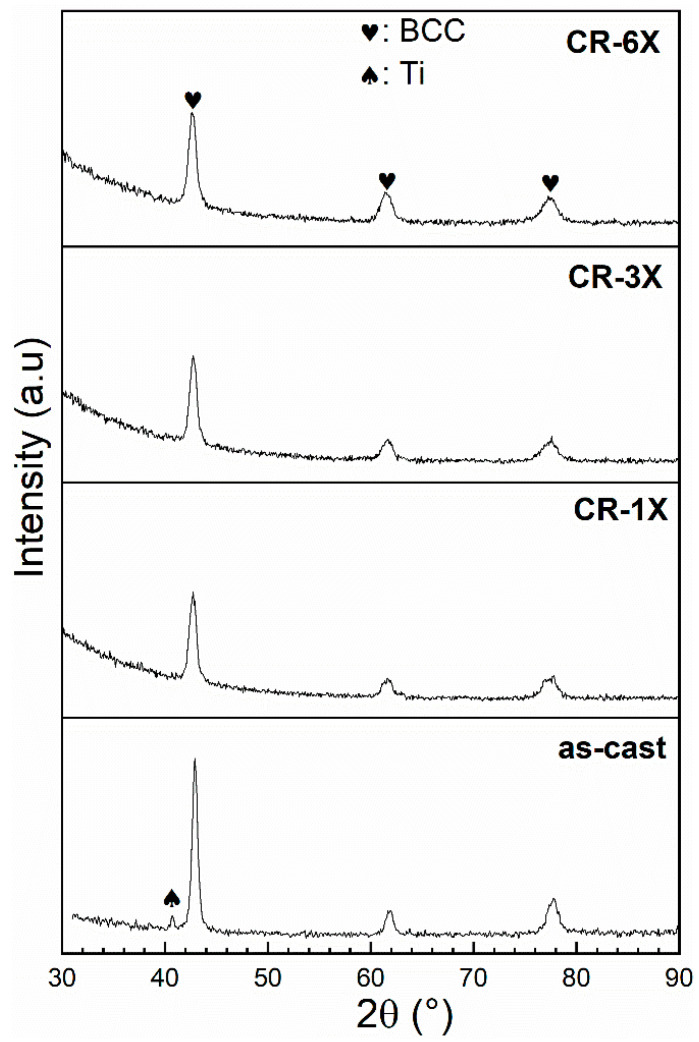
XRD patterns of the as-cast, CR-1X, CR-3X, and CR-6X of Ti_1_V_0.9_Cr_1.1_ alloy.

Figure 2 presents the XRD patterns of the as-cast Ti_1_V_0.9_Cr_1.1_ alloy and after 1X, 3X, and 6X CR. All patterns showed a BCC structure (S.G. *Im-3m*). The crystal structure parameters and the weighted profile factor R_wp_ as evaluated by Rietveld’s refinement, are presented in Table 1.

As seen in Table 1, crystallite size did not drastically change upon rolling, whereas the microstrain slightly increased but only for the first rolling. Further rolling did not change the microstrain. The disappearance of the Ti-phase in the CR patterns after rolling occurred because the crystallite size of this phase tended to decrease and the microstrain increased. These two factors caused the peak to broaden. As the Ti phase was already at the limit of detection in the as-cast pattern (the value was 3 ± 3%), as the peaks broadened, they became undistinguishable from the background.

### 3.3. First Hydrogenation

**Figure 3:**
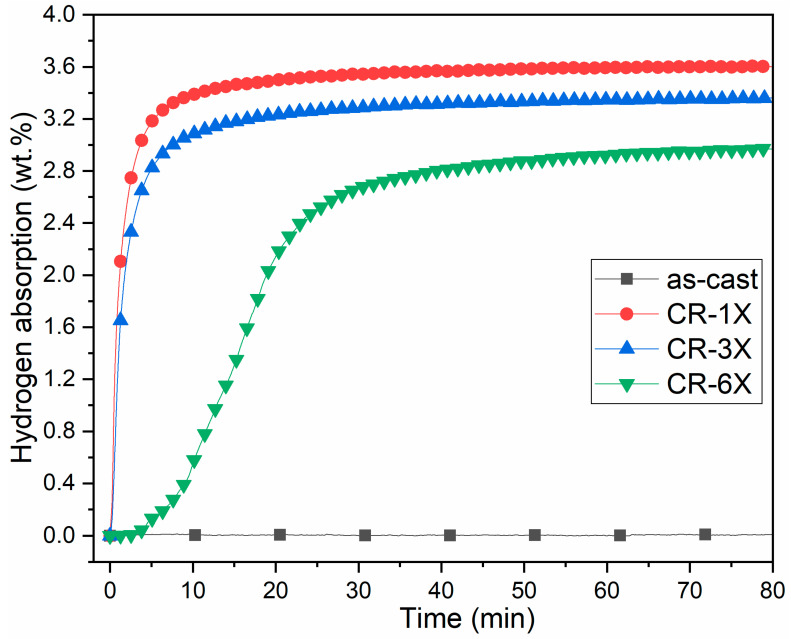
Activation curves of the as-cast, CR-1X, CR-3X, and CR-6X of Ti_1_V_0.9_Cr_1.1_ alloy.

The first hydrogenation (activation) of the as-cast and cold rolled alloys was performed at room temperature under 20 bars of hydrogen and without any prior heat treatment. The activation kinetics are shown in Figure 3.

The as-cast Ti_1_V_0.9_Cr_1.1_ alloy is hard to activate and did not absorb hydrogen even after 15 h of hydrogen exposure. Just one pass of CR made the activation possible without any incubation time and with fast kinetics. Full capacity was reached after only 12 min. The CR-3X sample also demonstrated good hydrogen uptake but with a slight reduction in capacity. Further rolling to 6X showed a 4 min incubation time followed by a slower absorption and reduced total capacity. The effectiveness of CR in activating the Ti_1_V_0.9_Cr_1.1_ alloy is still unclear. Considering the crystal structure before and after CR, one rolling pass did not change the crystal structure of the alloy, and the reduction in crystallite size was relatively small. Therefore, the reason is something else. CR breaks particles in smaller pieces, thus producing new surfaces that could be active to hydrogen. However, as CR was performed in air, the newly produced surfaces would, in principle, oxidize immediately. Nevertheless, the speed of oxidation could be so slow that the freshly produced surfaces have only very thin oxide, which could be easily broken during hydrogenation. Figure 3 shows that the capacity after 1, 3, and 6 CR passes are 3.6, 3.3, and 2.9 wt %, respectively. Therefore, the loss of capacity appears to be directly proportional to the number of rolling passes. Each rolling pass decreased the capacity by about 0.1 wt %. This decrease is most likely the formation of oxide as the rollings were performed in air.

**Figure 4:**
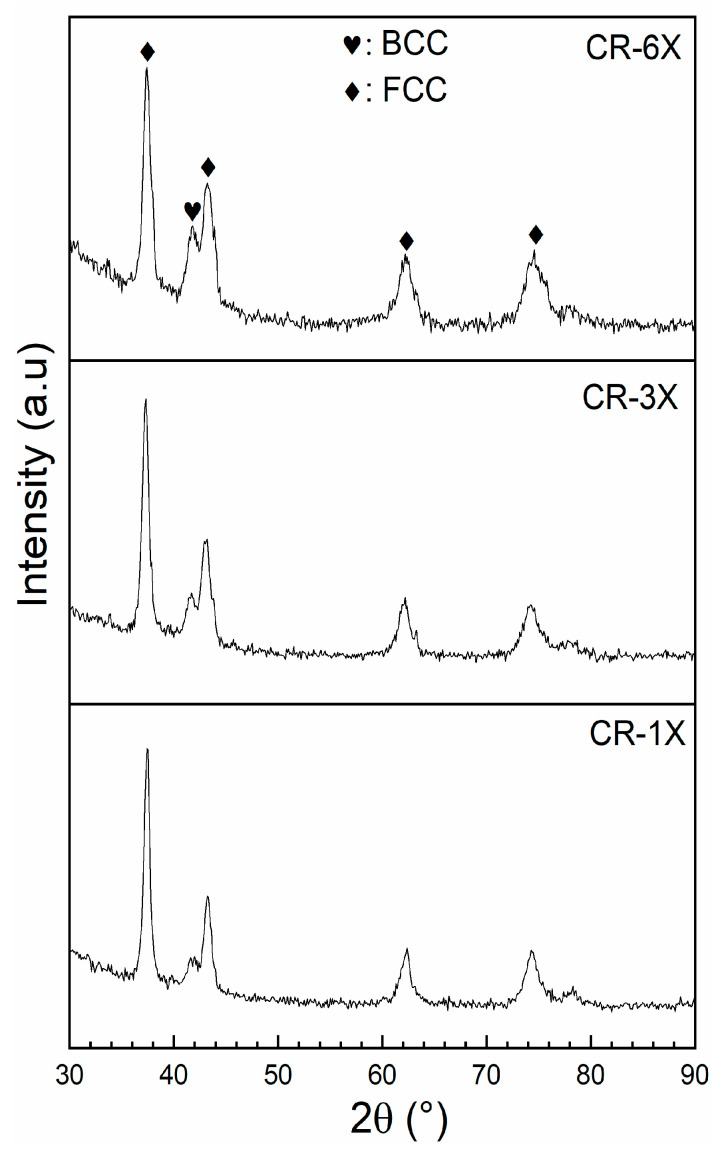
X-ray diffraction (XRD) patterns of hydrogenated of CR-1X, CR-3X, and CR-6X of Ti_1_V_0.9_Cr_1.1_ alloy.

The crystal structure of the full hydride samples was investigated from XRD patterns. Figure 4 presents the XRD patterns of the hydride samples of CR-1X, CR-3X, and CR-6X of Ti_1_V_0.9_Cr_1.1_ alloy.

Figure 4 clearly shows that BCC and FCC phases were present in all patterns, but the relative intensities of both phases are different. Despite the capacity decreasing with increasing number of rolling passes, no oxide phase was identified in these patterns. For the 6X pattern, the proportion of oxide should be approximately 17 wt % and should be seen in the diffraction pattern. The absence of the oxide phase in the patterns may be due to the very small crystallite size of the oxide phase. Assuming that the oxide phase is a shell around the hydride phase, the oxide’s thickness is about 6% of the radius of the hydride crystallite. As seen in Table 2, which shows the results of Rietveld’s refinement of all hydride patterns, the crystallite size of the FCC hydride phase is 30 nm; this means that the thickness of the oxide phase could be as small as 0.8 nm. Such a small crystallite size produces a diffraction pattern with very broad peaks that are effectively indistinguishable from the background.

The abundance of the phase (FCC) decreased with the number of rolls, and this seems to agree with the decreases in capacity shown in Figure 3. All the hydride patterns had BCC and FCC phases with almost the same lattice parameters. However, the lattice parameter of the BCC phase was much bigger than the as-cast BCC phase reported in Table 1. This was an indication that the BCC phase also contained hydrogen. The volume expansion of the BCC phase was about 2.6 Å^3^. Considering that a hydrogen atom occupies a volume between 2 and 3 Å^3^, and that there are two lattice points per unit cell, we estimated that the BCC phase contained about 0.5 hydrogen atom per metallic atom (H/M ≈ 0.5). Since the diffraction patterns were captured in air at room temperature, it is possible that the samples experienced a partial dehydrogenation. To determine the true crystal structure in the fully hydrided state, an in-situ diffraction pattern should be recorded.

### 3.4. Effect of Ball Milling on Ti_1_V_0.9_Cr_1.1_ Alloy

**Figure 5:**
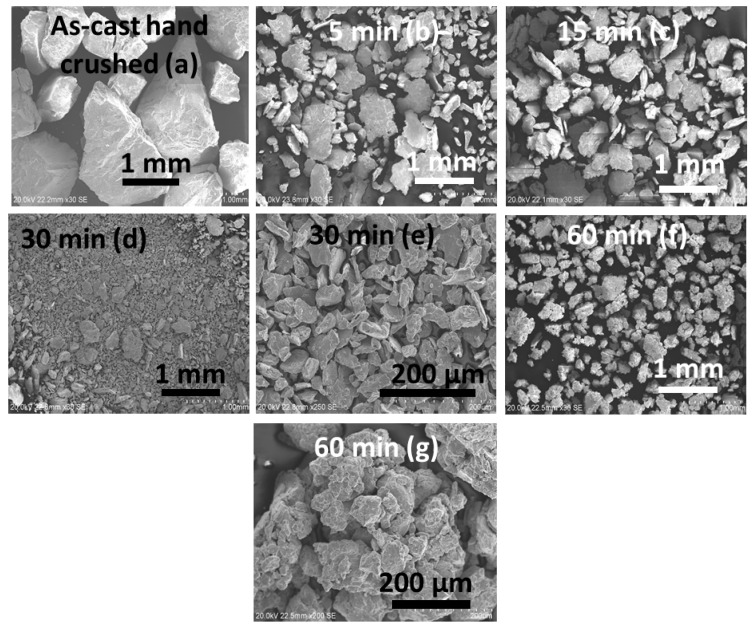
SEM micrographs of Ti_1_V_0.9_Cr_1.1_ alloy before (**a**) and after 5 (**b**), 15 (**c**), 30 (**d**,**e**), and 60 min (**f**,**g**) of ball milling (BM).

As CR was found to be beneficial for activation, we wanted to determine if BM produces the same effect. For this, the as-cast alloy was milled for 5, 15, 30, and 60 min. The morphologies of the processed powders are shown in Figure 5.

After 5 min BM, some flat surfaces and formation of smaller particles in the range 0.2–1 mm were observed. Further milling decreased the size of the particles. Agglomeration of small particles was observed after 60 min of milling.

**Figure 6:**
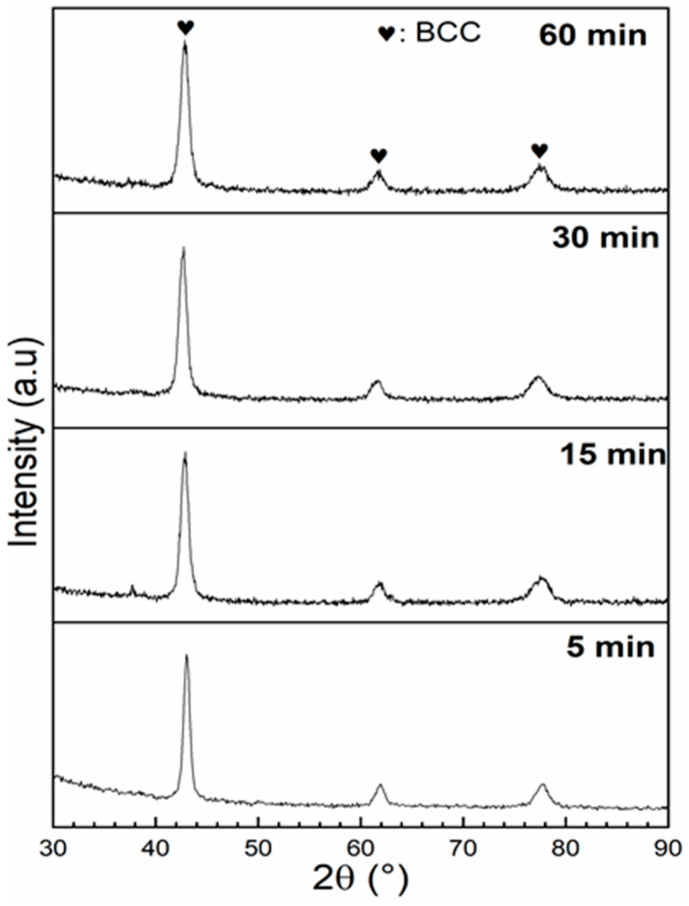
Diffraction patterns of Ti_1_V_0.9_Cr_1.1_ alloy for different BM times.

The XRD patterns of Ti_1_V_0.9_Cr_1.1_ alloy for different BM times are presented in Figure 6. The crystal structure parameters and the R_wp_, as evaluated by Rietveld’s refinement, are shown in Table 3.

All the ball-milled samples had a BCC crystal structure. The crystallite size decreased with milling while the microstrain remained almost constant. The microstrain in the ball-milled samples was almost identical to that in the cold rolled samples.

**Figure 7:**
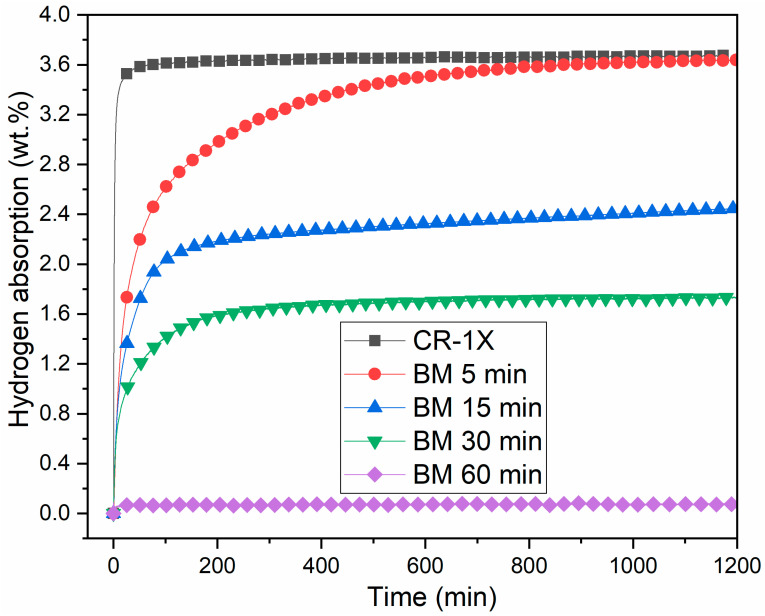
Activation curves of Ti_1_V_0.9_Cr_1.1_ alloy for different BM times as compared to the 1 cold-rolled sample.

The activation curves of the ball-milled samples compared to the cold-rolled sample are presented in Figure 7.

BM enabled the activation of Ti_1_V_0.9_Cr_1.1_ alloy. Milling for 5 min significantly improved the kinetics with a maximum capacity of 3.6 wt % H_2_. Further milling for 15 and 30 min reduced the capacity to 2.4 and to respectively 1.7 wt % of hydrogen. Milling for one hour made the sample completely inert to hydrogen. This behavior is still unclear and suggests that, besides the sample’s morphology, other parameters impact the activation. The effect of particle size in a BCC alloy was investigated by Kamble et al. [15]. For the first hydrogenation, they found that particle size affects the incubation time but has no significant effect on the capacity. Lou et al. found similar results on BCC V_40_(TiCr)_51_Fe_8_Mn alloy [36]. In their study, the particle size was 60 to 500 mesh (0.25 to 0.025 mm) but they also registered the same hydrogen capacity in the first cycle with a different loss of capacity with cycling. After 25 cycles, the best performance was obtained for the 400-mesh sample, which lost 11% of its initial capacity.

Comparing the result of CR-1X with that of BM-5 min, both are beneficial for activation of the Ti_1_V_0.9_Cr_1.1_ alloy, but CR-1X is more effective since full hydrogenation occurs much faster.

In a previous investigation, we showed that an addition of 12 wt % Zr to Ti_1_V_0.9_Cr_1.1_ alloy produced fast activation kinetics, reaching a maximum capacity of 3 wt % H_2_ within 3 min [14]. Therefore, adding 12 wt % Zr still produces faster kinetics compared to CR-1X but with reduced capacity. However, using mechanical deformation such as BM and CR does not change the chemistry. Adding other elements may increase the cost of the alloy and change the chemistry of the BCC phase. For these reasons, activation enhancement is better achieved by mechanical than by additive treatment.

**Figure 8:**
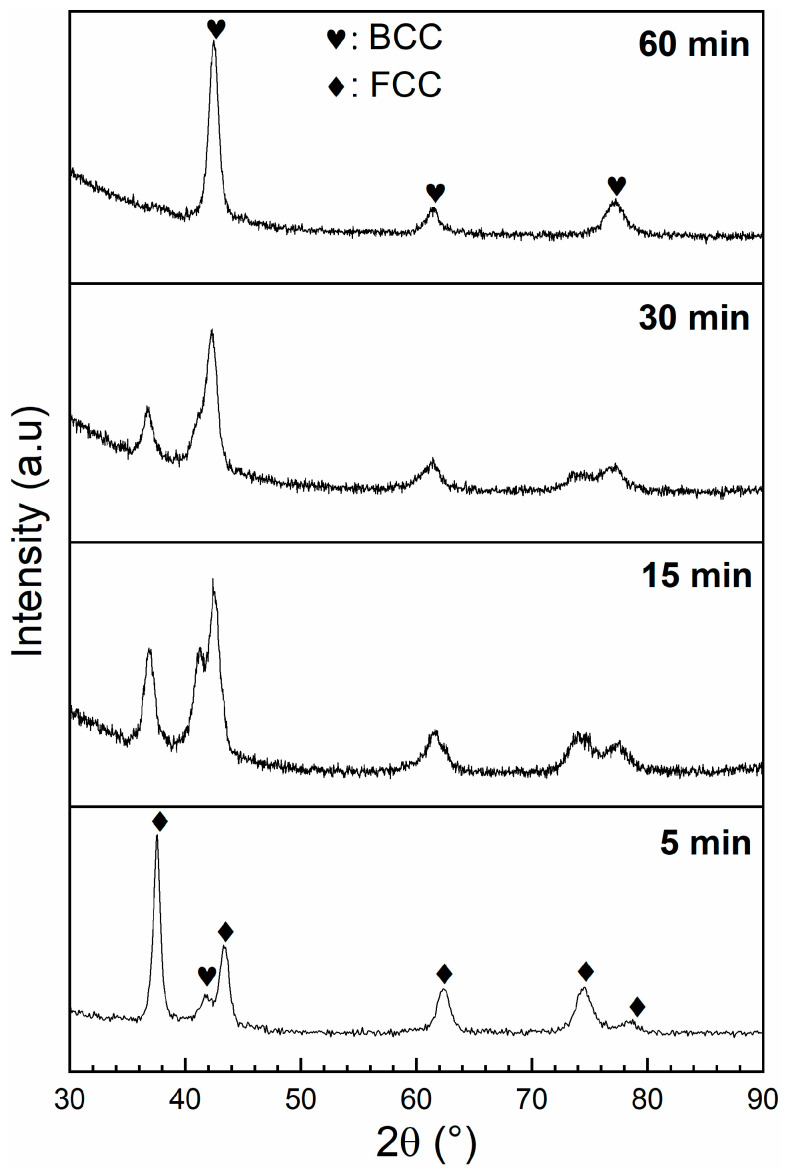
Diffraction patterns of Ti_1_V_0.9_Cr_1.1_ alloy for different BM times after hydrogenation.

We observed that the hydrogenation was complete for all BM samples, even if the total capacity was reduced. To determine the crystal structure of the ball-milled samples after hydrogenation, we performed XRD patterns, as seen in Figure 8. The crystal structure parameters and the R_wp_ values as determined by Rietveld refinement, are shown in Table 4.

For the 60 min sample, the crystal structure was still BCC, which confirmed that this sample did not absorb hydrogen, as seen in Figure 7. There was no significant change in the lattice parameter and crystallite size of this BCC phase, confirming that there was no reaction with hydrogen. All the other hydride patterns had BCC and FCC phases. The abundance of the FCC phase decreased with milling time, which agrees with the reduction of hydrogen capacity seen in Figure 7. The unit cell volume of the FCC phase was practically identical to the FCC phase observed in the hydrogenated cold-rolled samples. However, the unit cell volume of the BCC phase showed different behavior. It progressively decreased with milling time; this means that as milling time increased, the amount of hydrogen retained in the BCC phase decreased. It seems that milling decreases the ability of the BCC phase to absorb hydrogen. The exact mechanism is not clear, but it is a subject for further research and may explain the reduction of capacity with milling time.

## 4. Conclusions

In this paper, the effects of CR and BM on the microstructure and hydrogen storage properties of Ti_1_V_0.9_Cr_1.1_ alloy were reported. Cold-rolled samples for one, three, and six rolling passes had a BCC crystal structure. One cold roll was efficient for the first absorption kinetics. However, increasing rolling passes led to a reduction in capacity. The BCC and FCC phases were present in the cold-rolled samples after hydrogenation. Ball-milled samples for 5, 15, 30, and 60 min also maintained the original BCC crystal structure, and no other crystal structure was identified. After BM for five minutes, the first hydrogenation proceeded with relatively good kinetics. However, further milling decreased hydrogen capacity. Milling to 60 min made the alloy inert to hydrogen. The hydrogenated samples had both BCC and FCC phases.

One cold roll and BM for five minutes were beneficial for the first hydrogenation kinetics. CR-1X was more effective than ball milling for five minutes since full hydrogenation occurred much faster. The exact mechanism responsible for the improvement of activation kinetics upon CR and BM is still unknown. Mechanical treatment, and especially CR, are more efficient, low-cost, and improve activation kinetics more than addition of other elements such as Zr.

## Figures and Tables

**Table 1:** Crystal structure parameters and the R_wp_ values of the as-cast, CR-1X, CR-3X, and CR-6X of Ti_1_V_0.9_Cr_1.1_ alloy. Error on the last significant digit is indicated in parentheses.

Sample	Phase	Unit Cell Volume (Å^3^)	Lattice Parameter (Å)	Crystallite Size (nm)	Microstrain (%)	Phase Abundance (%)	R_wp_
As-cast	BCC Ti	28.04 (2) 36.25 (1)	3.0379 (9) a = 2.961 (4) c = 4.773 (1)	24 (2) 13 (2)	0.26 (1)	97 (3) 3 (3)	7.76
CR-1X	BCC	28.24 (3)	3.0453 (1)	28 (5)	0.40 (1)	100	5.95
CR-3X	BCC	28.34 (4)	3.0487 (1)	25 (4)	0.40 (1)	100	6.07
CR-6X	BCC	28.26 (4)	3.0459 (1)	21 (3)	0.42 (2)	100	6.55

**Table 2:** Crystal structure parameters and the R_wp_ values of all patterns after hydrogenation. Error on the last significant digit is indicated in parentheses.

Sample	Phase	Unit Cell Volume (Å^3^)	Lattice parameter (Å)	Crystallite Size (nm)	Microstrain (%)	Phase Abundance (%)	R_wp_
CR-1X	FCC BCC	79.03 (1) 30.84 (8)	4.2914 (2) 3.136 (3)	20.2 (2) 7 (2)	0.34 (1) 0.43 (1)	84 (2) 16 (2)	5.34
CR-3X	FCC BCC	78.90 (1) 30.65 (9)	4.289 (2) 3.130 (7)	17.3 (1) 4.0 (6)	0.40 (1) 0.33 (1)	76 (3) 24 (3)	5.28
CR-6X	FCC BCC	78.61 (1) 30.67 (7)	4.284 (2) 3.130 (2)	29 (6) 4.9 (6)	0.53 (2) 0.36 (8)	72 (2) 28 (2)	4.93

**Table 3:** Crystal parameters and the R_wp_ values of Ti_1_V_0.9_Cr_1.1_ alloy for different ball milling times compared with the as-cast sample. Error on the last significant digit is indicated in parentheses.

Sample	Phase	Unit Cell Volume (Å^3^)	Lattice Parameter (Å)	Crystallite Size (nm)	Microstrain (%)	Phase Abundance (%)	R_wp_
As-cast	BCC Ti	28.04 (2) 36.25 (1)	3.0379 (9) a = 2.961(4) c = 4.773 (1)	24 (2) 13 (2)	0.26 (1)	97 (3) 3 (3)	7.76
BM 5 min	BCC	28.29 (3)	3.0471 (9)	20.5 (1)	0.30 (1)	100	5.20
BM 15 min	BCC	28.18 (3)	3.0432 (1)	16.1 (1)	0.40 (2)	100	7.84
BM 30 min	BCC	28.28 (3)	3.0468 (1)	14.5 (8)	0.40 (1)	100	7.46
BM 60 min	BCC	28.32 (3)	3.0481 (1)	12.2 (6)	0.40 (1)	100	7.30

**Table 4:** Crystal parameters and the R_wp_ values of Ti_1_V_0.9_Cr_1.1_ alloy for different ball milling times after hydrogenation. Error on the last significant digit is indicated in parentheses.

Activated BM	Phase	Unit Cell Volume (Å^3^)	Lattice Parameter (Å)	Crystallite Size (nm)	Microstrain (%)	Phase Abundance (%)	R_wp_
5 min	FCC BCC	78.97 (7) 31.06 (7)	4.2903 (1) 3.144 (2)	15.4 (8) 3.8 (5)	0.35 (1) 0.34 (1)	79 (2) 21 (2)	4.12
15 min	FCC BCC	78.38 (1) 29.33 (5)	4.280 (2) 3.0841 (2)	15.8 (2) 8.6 (1)	0.42 (2) 1.09 (4)	36 (2) 64 (2)	5.12
30 min	FCC BCC	78.49 (2) 28.75 (5)	4.282 (3) 3.0634 (2)	14.0 (2) 5.1 (3)	0.29 (3) 0.49 (3)	23 (2) 77 (2)	5.17
60 min	BCC	28.15 (3)	3.0418 (9)	12.4 (6)	0.40 (1)	100	4.63

## References

[1] Von Colbe J.B., Ares J.-R., Barale J., Baricco M., Buckley C., Capurso G., Gallandat N., Grant D.M., Guzik M.N., Jacob I. (2019). Application of hydrides in hydrogen storage and compression: Achievements, outlook and perspectives. Int. J. Hydrogen Energy.

[2] Rusman N.A.A., Dahari M. (2016). A review on the current progress of metal hydrides material for solid-state hydrogen storage applications. Int. J. Hydrogen Energy.

[3] Shelyapina M.G., Martínez L.M.T., Kharissova O.V., Kharisov B.I. (2017). Metal hydrides for energy storage. Handbook of Ecomaterials.

[4] Mazzolai G., Coluzzi B., Biscarini A., Mazzolai F.M., Tuissi A., Agresti F., Lo Russo S., Maddalena A., Palade P., Principi G. (2008). Hydrogen-storage capacities and H diffusion in bcc TiVCr alloys. J. Alloys Compd..

[5] Miraglia S., de Rango P., Rivoirard S., Fruchart D., Charbonnier J., Skryabina N. (2012). Hydrogen sorption properties of compounds based on BCC Ti_1−*x*_V_1−*y*_Cr_1+*x*+*y*_ alloys. J. Alloys Compd..

[6] Tamura T., Kazumi T., Kamegawa A., Takamura H., Okada M. (2003). Protium absorption properties and protide formations of Ti–Cr–V alloys. J. Alloys Compd..

[7] Kuriiwa T., Maruyama T., Kamegawa A., Okada M. (2010). Effects of V content on hydrogen storage properties of V–Ti–Cr alloys with high desorption pressure. Int. J. Hydrogen Energy.

[8] Selvaraj S., Jain A., Kumar S., Zhang T., Isobe S., Miyaoka H., Kojima Y., Ichikawa T. (2018). Study of cyclic performance of V-Ti-Cr alloys employed for hydrogen compressor. Int. J. Hydrogen Energy.

[9] Shelyapina M.G., Skryabina N.E., Surova L.S., Dost A., Ievlev A.V., Privalov A.F., Fruchart D. (2019). Proton NMR study of hydrogen mobility in (TiCr_1.8_)_1−x_V_x_ hydrides. J. Alloys Compd..

[10] Lin H.C., Lin K.M., Wu K.C., Hsiung H.H., Tsai H.K. (2007). Cyclic hydrogen absorption–desorption characteristics of TiCrV and Ti_0.8_Cr_1.2_V alloys. Int. J. Hydrogen Energy.

[11] Itoh H., Arashima H., Kubo K., Kabutomori T. (2002). The influence of microstructure on hydrogen absorption properties of Ti–Cr–V alloys. J. Alloys Compd..

[12] Huot J., Léon A. (2008). Kinetics and thermodynamics. Hydrogen Technology: Mobile and Portable Applications.

[13] Hirose K. (2010). Handbook of Hydrogen Storage: New Materials for Future Energy Storage.

[14] Sleiman S., Huot J. (2017). Microstructure and Hydrogen Storage Properties of Ti_1_V_0.9_Cr_1.1_ Alloy with Addition of *x* wt % Zr (*x* = 0, 2, 4, 8, and 12). Inorganics.

[15] Kamble A., Sharma P., Huot J. (2017). Effect of doping and particle size on hydrogen absorption properties of BCC solid solution 52Ti-12V-36Cr. Int. J. Hydrogen Energy.

[16] Bellon D., Martinez A., Barreneche D., dos Santos D. (2016). A structural study of the hydrogen absorption properties by replacing vanadium with zirconium in metal alloys. J. Phys. Conf. Ser..

[17] Shelyapina M., Dost A., Skryabina N., Privalov A., Vogel M., Fruchart D. (2020). Effect of Zr_7_Ni_10_ additive on hydrogen mobility in (TiCr_1.8_)_1−*x*_V*_x_* (*x* = 0.2, 0.4, 0.6, 0.8): An ^1^H NMR SFG study. Int. J. Hydrogen Energy.

[18] Skryabina N.E., Fruchart D., Medvedeva N.A., de Rango P., Mironova A.A. (2017). Correlation between the Hydrogen Absorption Properties and the Vanadium Concentration of Ti-V-Cr Based Alloys. Solid State Phenomena.

[19] Kamble A.G., Sharma P., Huot J. (2019). Investigation of crystal structure, microstructure and hydrogenation behavior of heat-treated Ti_52_V_12_Cr_36_ alloy. ACS Appl. Energy Mater..

[20] Zhou H.Y., Wang F., Wang J., Wang Z.M., Yao Q.R., Deng J.Q., Tang C.Y., Rao G.H. (2014). Hydrogen storage properties and thermal stability of V_35_Ti_20_Cr_45_ alloy by heat treatment. Int. J. Hydrogen Energy.

[21] Rong M., Wang F., Wang J., Wang Z., Zhou H. (2017). Effect of heat treatment on hydrogen storage properties and thermal stability of V_68_Ti_20_Cr_12_ alloy. Prog. Nat. Sci. Mater. Int..

[22] Couillaud S., Enoki H., Amira S., Bobet J.-L., Akiba E., Huot J. (2009). Effect of ball milling and cold rolling on hydrogen storage properties of nanocrystalline TiV_1.6_Mn_0.4_ alloy. J. Alloys Compd..

[23] Huot J., Enoki H., Akiba E. (2008). Synthesis, phase transformation, and hydrogen storage properties of ball-milled TiV_0.9_Mn_1.1_. J. Alloys Compd..

[24] Singh B.K., Shim G., Cho S.-W. (2007). Effects of mechanical milling on hydrogen storage properties of Ti_0.32_Cr_0.43_V_0.25_ alloy. Int. J. Hydrogen Energy.

[25] Varin R.A., Czujko T., Wronski Z.S. (2009). Nanomaterials for Solid State Hydrogen Storage.

[26] Sleiman S., Huot J. Effect of particle size, pressure and temperature on the activation mechanism of hydrogen absorption in TiVZrHfNb high entropy alloy.

[27] Shelyapina M., Fruchart D., De Rango P., Charbonnier J., Rivoirard S., Skryabina N., Miraglia S., Hlil E., Wolfers P. (2006). First-Principles Investigation of the Stability of the Ti-V-Cr Ternary Alloys and Their Related Hydrides. AIP Conference Proceedings.

[28] Huot J. (2016). Enhancing Hydrogen Storage Properties of Metal Hybrides: Enhancement by Mechanical Deformations.

[29] Vega L.E.R., Leiva D.R., Leal Neto R.M., Silva W.B., Silva R.A., Ishikawa T.T., Kiminami C.S., Botta W.J. (2018). Mechanical activation of TiFe for hydrogen storage by cold rolling under inert atmosphere. Int. J. Hydrogen Energy.

[30] De Araujo-Silva R.A., Jorge A.M., Vega L.E.R., Leal Neto R.M., Leiva D.R., Botta W.J. (2019). Hydrogen desorption/absorption properties of the extensively cold rolled β Ti–40Nb alloy. Int. J. Hydrogen Energy.

[31] Tousignant M., Huot J. (2014). Hydrogen sorption enhancement in cold rolled LaNi_5_. J. Alloys Compd..

[32] Patselov A., Rybin V., Greenberg B., Mushnikov N. (2010). Hydrogen absorption in as-cast bcc single-phase Ti–Al–Nb alloys. J. Alloys Compd..

[33] Khajavi S., Rajabi M., Huot J. (2019). Effect of cold rolling and ball milling on first hydrogenation of Ti_0.5_Zr_0.5_ (Mn_1−x_Fe_x_) Cr_1_, x = 0, 0.2, 0.4. J. Alloys Compd..

[34] Manna J., Tougas B., Huot J. (2018). Mechanical activation of air exposed TiFe + 4 wt% Zr alloy for hydrogenation by cold rolling and ball milling. Int. J. Hydrogen Energy.

[35] Bruker AXS (2017). Topas V6: General Profile and Structure Analysis Software for Powder Diffraction Data—User’s Manual.

[36] Luo L., Wu C., Yang S., Zhou J., Chen Y., Yang F., Xu Y., Liu P. (2015). Decaying behaviors of V_40_(TiCr) _51_Fe_8_Mn hydrogen storage alloys with different particle sizes. J. Alloys Compd..

